# Development of an *in vitro* drug sensitivity assay based on newly excysted larvae of *Echinostoma caproni*

**DOI:** 10.1186/1756-3305-6-237

**Published:** 2013-08-13

**Authors:** Gordana Panic, Katrin Ingram, Jennifer Keiser

**Affiliations:** 1 Department of Medical Parasitology and Infection Biology, Swiss Tropical and Public Health Institute, PO Box, Basel, CH-4002, Switzerland; 2 University of Basel, Basel, CH-4003, Switzerland

**Keywords:** *Echinostoma caproni*, Echinostomiasis, Excystation, Metacercariae, Drug sensitivity assay, *In vitro*, Chemotherapy

## Abstract

**Background:**

Echinostomiasis is one of the major food-borne trematodiases and the species *Echinostoma caproni* serves as a useful model for trematocidal drug discovery. The current *in vitro* drug sensitivity assay uses adult *E. caproni* worms that are incubated with candidate drugs and scored microscopically for viability at 72 hrs. The aim of this study was to investigate the use of newly excysted larvae (NEL) of *E. caproni* for *in vitro* drug testing, which would be faster, more cost effective and more ethical compared to adult worm assays.

**Methods:**

Larvae were obtained by collecting metacercariae from snails and triggering their excystation using the trypsin-bile salt excystation method. Studies concerning various parameters of this chemical transformation process as well as appropriate NEL culturing conditions were carried out and findings evaluated. NEL and adult worms were incubated with praziquantel, tribendimidine, albendazole and quinine and evaluated microscopically 72 hrs post-incubation. In addition, the colorimetric markers resazurin, CellTiter-Glo® and Vybrant® were tested as an alternative assay read-out method.

**Results:**

The chemical excystation method successfully induced *E. caproni* metacercariae to excyst at a rate of about 20-60%. NEL remained viable in culture medium for 5–7 days. The results of an *in vitro* drug assay using NEL mirrored the results of an assay using adult worms incubated with the same drugs. None of the markers could reliably produce signals proportional to NEL viability or cytotoxicity without significant complications.

**Conclusion:**

NEL are adequate for *in vitro* drug testing. Challenges remain in further improving the excystation yield and the practicability of the assay setup. Resolving these issues could also improve read-outs using colorimetric markers. Using NEL is in alignment with the 3 R rules of the ethical use of laboratory animals and can greatly increase the rate and affordability with which drugs are screened *in vitro* against this intestinal trematode.

## Background

Echinostomiasis is a major food-borne intestinal trematodiasis caused by a number of *Echinostoma* species acquired by the ingestion of uncooked freshwater fish, crustaceans, snails or amphibians. It is a focal disease found in Southeast Asia and the Far East usually in poor rural areas and is associated with poor sanitary conditions, poor economic conditions and malnutrition [1]. Prevalence, Disability-Adjusted Life Years (DALYs) and economic impact can be difficult to assess due to the lack of quality surveys and reporting [2]. Recently, the first estimates on the burden of food-borne trematodiases have been established and it has been calculated that intestinal flukes overall contribute to 83 699 DALYs [3]. Infections are most effectively treated by praziquantel, administered at 10–25 mg/kg body weight in a single oral dose [4,5].

*E. caproni* is a convenient model for studies on food-borne trematodes. Its life-cycle is easy to maintain in the laboratory, involving only a mouse as the definitive host and *Biomphalaria glabrata* snails as the intermediate host [6]. Both *in vitro* and *in vivo* studies with *E. caproni* can be conducted with relative ease, as mice can be infected with 30 to almost 300 metacercariae and an adult infection is established as soon as 2 weeks post-infection [6]. *E. caproni* can also be used as a pre-screening tool for potential trematocidal compounds, though caution must be applied, as *E. caproni* has different feeding habits and niches in the definitive host as compared to other trematodes [7].

Currently, *in vitro* drug testing with *E. caproni* is conducted with the adult stage of the worm [8]. The adult worms are incubated in the presence of candidate drugs and monitored at selected time points. Their viability is assessed via microscopic read-out, by observing the morphology and motility of the worms. Though the current *E. caproni* model is an effective drug discovery tool, the *in vitro* screens using adult *E. caproni* worms are disadvantageous in that they require the sacrifice of mice, they yield a small quantity of worms and they are time-consuming and expensive. An *in vitro* assay using newly excysted larvae (NEL) would yield more worms for testing, would be cheaper and faster and would be better aligned with the 3Rs animal protection principles (reduce, replace, refine), by replacing adult worms obtained from sacrificed mice, with NEL. Additionally, replacing microscopic read-out with an automated read-out using colorimetric markers could increase the efficiency and objectivity of the screens.

The aim of the present study was to develop an *in vitro* drug sensitivity assay based on NEL. First, different parameters based on a previously described *in vitro* excystation method were tested and optimized [9]. Second, NEL were cultured in various media to determine optimal culturing conditions. Subsequently, a NEL-based drug sensitivity assay was set up and evaluated against the gold standard, a drug sensitivity assay using adult *E. caproni* worms. In addition, 3 colorimetric markers were tested in a NEL-based assay as alternative read-out methods.

## Methods

### Drugs, chemicals and media

Praziquantel, quinine and albendazole were purchased from Sigma-Aldrich (Buchs, Switzerland), and tribendimidine was donated by the Shandong Xinhua Pharmaceutical Company (Zibo, China). Drug stock solutions were made by dissolving the compounds in DMSO (dimethyl sulfoxide, Fluka, Buchs, Switzerland) at a concentration of 10 mg/ml and were stored at −20°C until use.

RPMI 1640, Medium 199 (Med 199), Dulbeco’s modified eagle medium (DMEM) and Minimum essential medium (MEM) were purchased from Gibco (Basel, Switzerland). Phosphate Buffer Solution (PBS), Amphotericin B (250 μg/ml), penicillin-streptomycin (10 000 units penicillin and 10 mg/mL streptomycin), bile salts (B8756), trypsin 10× solution (T4549) and L-cysteine (168149) were purchased from Sigma-Aldrich (Buchs, Switzerland).

RPMI medium was put together as described by Ingram and colleagues [10]. Locke’s 1:1 solution was prepared according to studies conducted by Ursone and Fried and adjusted to pH 7.4 [11]. Basch medium was made as described previously [12].

The CellTiter-Glo® viability assay kit was purchased from Promega and the Vybrant® Cytotoxicity Assay Kit was purchased from Invitrogen, whereas the resazurin marker was made by dissolving resazurin sodium salt (Sigma) in 1× PBS solution at a concentration of 125 mg/L. All markers were stored at –20°C until use.

### Parasites

The *E. caproni* life cycle was established at the Swiss Tropical and Public Health Institute in 2004 and has been maintained successfully since [6]. To obtain metacercariae for excystation experiments, the shells of infected snails were gently crushed and removed and a mass of cysts was located at the pericardial region of the snail. The cysts were sucked out and stored in Locke’s 1:1 solution in a 20 ml tube at 4°C until use.

### Excystation of *E. caproni* metacercariae into newly excysted larvae (NEL)

The excystation procedure used in this work was derived from Saxton *et al.*[9]. Briefly, cysts were treated with a trypsin-bile salt medium containing the reductant L-cysteine (hereon referred to as TBC medium). Specifically, 15 mg trypsin and 40 mg bile salts were diluted in 5 ml of NaCl/NaHCO_3_ solvent (8 g/L and 15 g/L respectively). In another tube, 40 mg of L-cysteine was diluted in 5 ml of 0.05 M HCl solvent. The two solutions were combined just before use and filtered. Around 25 cysts were placed in a well-plate, 3 ml of syringe-filtered TBC medium was added and the setup was placed in a hot water bath at 40°C. After 1 hr, the suspension was taken out, the number of excysted larvae was counted and an excystation percentage (or rate) was calculated. The TBC medium was removed and Locke’s 1:1 solution was added.

### Parameters affecting excystation rates

In order to maximize the excystation yield, experiments testing various parameters of the TBC excystation method were performed. For each experiment, the TBC excystation method was performed as described above, varying one variable at a time. The effects of the parameters were evaluated by calculating the excystation rate. For each experiment described below, values that are marked with * indicate the values in the original TBC excystation method [9] and thus served as control values.

#### Cyst storage conditions

The cyst storage time affects cyst viability [13] and, therefore, their excystation rate. The effect of cyst storage time on their viability was monitored over 6 weeks. The TBC excystation was applied on a sampling of the cysts each week for up to 6 weeks. Storage medium was changed once every 2 weeks. Cyst viability was determined on the basis of how many cysts excysted in each treatment group. Due to frequent fungal contaminations of the medium the effect of adding 2% Amphotericin B solution to the storage medium was also tested.

#### Trypsin, bile salts and L-cysteine concentration

The effect of trypsin, bile salt and L-cysteine concentrations in the TBC medium were assessed over a range of concentrations: 0, 1, 1.5*, 3 and 5 mg/ml for trypsin, 0, 3, 4*, 5 and 7 mg/ml for bile salts and 0, 2, 4*, 8 and 40 for L-cysteine.

#### TBC medium pH, temperature and incubation time

In separate experiments, the optimal pH range of the TBC medium, incubation temperature and incubation time were determined. Cysts were incubated in TBC medium at a pH of 7, 7.5, 7.8, 7.9, 8.0, 8.1, 8.2, 8.5 or 9. The incubation temperatures tested were 35, 37, 38, 39, 40*, 41, 42, 43 and 45°C and the length of exposure to TBC medium was tested at 1*, 1.5, 2, 2.5, 3, 4, 6 and 24 hrs.

#### Cyst number and volume of TBC medium

The effect of varying the amount TBC medium added (1, 2, 3*, 4, 5 and 10 ml) as well as the cyst amount (25*, 40, 60, 80 and 100 cysts) was elucidated.

#### Acid-pepsin pre-treatment

Other species of echinostomes require an acid-pepsin pre-treatment [14,15] and thus this was attempted here. Cysts were incubated in 3 ml of an acid-pepsin solution at 40°C for 2 hrs and then were treated with TBC medium as described above. The excystation rate of these cysts was compared to the excystation rate of cysts receiving only an acid pre-treatment or no pre-treatment.

### NEL culture conditions

The fitness of NEL in various culture media was tested. In a 96-well plate, 20 larvae were placed in 200 μL of either Locke’s 1:1 solution, RPMI, RPMI supplemented with 2% glucose, Basch, MEM, DMEM, PBS 1× or Med 199. Incubation tests in different media were conducted twice in duplicate. The viability of the worms was assessed microscopically at 24, 48, 72, 96 and 120 hrs using a viability scale as follows: 0 = dead; 1 = both impaired movement and markedly damaged tegument; 2 = slow movement or notable damage to tegument; and 3 = lively movement and undamaged tegument. Worms were scored individually and the scores averaged. Due to frequent fungal contaminations, the effect of adding 2% Amphotericin B solution to the culture medium was also tested.

### Drug assay with NEL

Four drugs were chosen for the assay: praziquantel, tribendimidine, albendazole and quinine. Praziquantel is the standard drug against echinostomiasis, tribendimidine has shown good activity against *E. caproni in vitro* and *in vivo*[8]*,* albendazole has previously been recommended for use [4] and quinine should not be effective and thus served as a negative control.

A serial dilution was prepared in a 96-well plate at concentrations of 30, 10, 3.3 1.1 and 0.37 μg/ml for tribendimidine, albendazole and quinine and at concentrations of 10, 3.3, 1.1, 0.37 and 0.12 μg/ml for praziquantel (due to previous observations of its high activity). The culture medium used was RPMI supplemented with 5% glucose and 1% penicillin/streptomycin mixture. Control wells consisted of culture medium with 1% DMSO. Drug dilutions were made in duplicate with a total volume of 200 μl, after which 50 μl of medium containing about twenty larvae were added per well. All experiments were conducted twice in duplicate.

The plate was read microscopically at 24, 48 and 72 hrs and the worms were scored according to the viability scale described above. After 72 hrs, NEL from the highest drug concentration wells were sampled and placed on a glass slide for microscopic observation. Microscopy was conducted using a Leica DM500B upright microscope (Solms, Germany) and images were taken using a Leica Application Suite camera and software (Solms, Germany).

### Drug assay with adult *E. caproni* worms

In order to compare results of the NEL assay to the current standard, a drug sensitivity assay was set up using adult worms and the same drugs and drug stock solutions. Adult *E. caproni* worms were obtained from dissected intestines of infected mice and were cultured in Med 199 supplemented with 1.5% glucose and 1% penicillin/streptomycin mixture. A serial dilution of the drugs was prepared in a 24-well plate at concentrations of 90, 30, 10, 3.3 and 1.1 μg/ml for tribendimidine, albendazole and quinine and of 30, 10, 3.3, 1.1 and 0.37 μg/ml for praziquantel. The total volume in each well was 2 ml. Control wells consisted of culture medium with 1% DMSO. Two worms were added per well and each drug was tested in duplicate. In total, 2 trials of 2 duplicates were performed. The plate was then read microscopically at 24, 48 and 72 hrs and the worms were scored according to the same viability scale used for NEL. At 72 hrs, worms in the wells containing 30 μg/ml of drug were observed using a Zeiss upright microscope (Oberkochen, Germany) and images were taken using a Canon PowerShot G10 camera (Tokyo, Japan).

### Colorimetric viability markers

Three colorimetric viability/cytotoxicity markers were assessed for their use in an NEL-based assay: resazurin, the CellTiter-Glo® Luminescent Viability Assay and the Vybrant® Cytotoxicity Assay Kit. Resazurin is a fluorescent viability marker measuring intracellular reducing capacity that has previously been successfully implemented in drug sensitivity screens against *Trypanosoma gambiense* and *Trypanosoma rhodesiense*[16] and with some success in screens against *Schistosoma mansoni*[17]. CellTiter-Glo® is a luminescent viability marker that measures intracellular ATP activity upon cell lysis and the Vybrant® cytotoxicity assay is a fluorescent assay that indirectly measures cell cytotoxicity by reacting with glucose 6-phosphate dehydrogenase (G6PD) that is released in the medium from dying cells.

An assay measuring the signal correlation to NEL number, as well as the optimal incubation time with the marker was set up for each of the 3 markers. For resazurin and CellTiter-Glo®, 20, 40, 60, 80 or 100 viable NEL were placed in duplicate wells in a 96-well plate topped up with up to 100 μl culture medium for CellTiter-Glo® and 200 μl culture medium for resazurin. Controls included 2 wells with medium only, 2 wells with 100 unexcysted metacercariae and 2 wells with 100 dead NEL. Dead NEL were killed by incubating the NEL overnight in culture medium containing 25% DMSO, then rinsing the suspension with culture medium. In the case of resazurin, 25 μl of resazurin solution was added to each well, incubated for 10 min and automatic read-outs were conducted at 10, 20, 30, 40, 50 and 60 min, and 2, 3, 4, 6 and 24 hrs. In the case of CellTiter-Glo®, 100 μl CellTiter-Glo® reagent was added and the assay was incubated for 10 min, then read at 10, 20, 30, 40, 50 and 60 min and 1.5 and 2 hrs. Vybrant® is a marker that measures cytotoxicity (cell death) rather than viability, thus 20, 40, 60, 80 and 100 dead (rather than viable) NEL were measured and 100 live NEL served as controls. The NEL were killed using the same method described above. The wells were topped up to 200 μl culture medium and subsequently 50 μl Vybrant® was added. The assay was incubated for 10 min and measurements were taken at 10, 20, 30, 40, 50, 60 min and 2, 3, 4, 6 and 24 hrs. All measurements were performed using the SpectraMax M2 plate reader (Molecular Devices, Sunnyvale, USA) and the corresponding SoftMax Pro® software.

### Statistics

Averages and standard deviations were calculated with Microsoft Office Excel 2003 or 2010. All graphs were generated in the same program. Additionally, a p-value, for significant difference between viability of cysts stored in Locke’s 1:1 versus Locke’s 1:1 supplemented with 2% Amphotericin B, was calculated using the student’s t-test, also using Microsoft Office Excel 2003 (unpaired, 2-tailed, assuming equal variance). The IC_50_ values and their corresponding r values of the NEL and adult drug sensitivity assays were calculated based on the median-effect principle using CompuSyn® software (ComboSyn Inc., 2007) [18]. The r-value represents the linear correlation coefficient and is a measurement of goodness of fit.

### Ethical approval

The work with *E. caproni* infected mice was approved by the veterinary authorities of the Canton Basel-Stadt (permit no. 2070) based on Swiss cantonal and national regulations.

## Results

### Parameters affecting excystation rates

The excystation rate of cysts stored in Locke’s 1:1 at 4°C remained at an average of 23.1 -25.7% for 3 weeks, after which it declined to 17.1% (± 2.4), 7.1% (± 5.7) and 4.0% (± 5.2) at 4, 5 and 6 weeks respectively. Moreover, the addition of 2% Amphotericin B to the storage medium significantly impaired excystation (t_16_ = 3.294, p = 0.005).

Varying the trypsin, bile salts and L-cysteine concentrations did not affect excystation rates, except that treatments where trypsin, bile salts or L-cysteine were omitted resulted in no excystation. Additionally, an excess of L-cysteine (40 mg/ml) also inhibited excystation.

The effects of temperature and pH of the TBC medium followed a bell curve. Excystation percentages were optimal at incubation temperatures of 39–41°C. At 35°C, no larvae excysted and at 45°C, although a minimal amount excysted, the NEL were damaged and impaired. Meanwhile, optimal excystment occurred at pH 7.8-8.2 in one trial, and at pH >7.8 in another trial.

A longer exposure time of the cysts to the TBC medium resulted in higher excystation rates. Excystation rates were stable at exposure times of 1–3 hrs, and started to markedly increase at 4 hrs. However, the long incubation time was detrimental to excysted larvae. Thus, an incubation time of 1.5-2.5 hrs was determined optimal, as longer incubation times started to negatively affect larval viability.

Finally, a variety of parameters showed no effect on metacercarial excystment. These include: the number of cysts added to the TBC medium, the volume of TBC medium applied to the cysts and pre-treatment of the cysts in an acid-only or an acid-pepsin solution.

### NEL culture conditions

**Figure 1:**
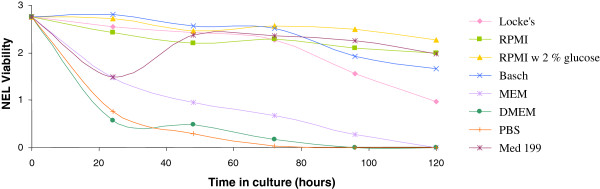
**Newly excysted larvae (NEL) incubated over time in various culture media.** Experiments are based on 2 trials performed in duplicate.

NEL remained viable for 5 days in Basch, Med 199, RPMI and RPMI with 2% glucose, whereas their viability declined moderately in Locke’s solution and sharply in PBS, MEM and DMEM (Figure 1). RPMI with 2% glucose performed slightly better than Basch, Med 199 and RPMI (though not significantly) and undocumented observations indicated that these larvae also often remained viable for up to 7 days.

### NEL drug sensitivity assay

IC_50_ values of praziquantel, tribendimidine, albendazole and quinine at 72 hrs post-exposure are presented in Table 1. Praziquantel and tribendimidine were highly active against NEL in that they were lethal within 24 hrs at a concentration of 30 μg/ml and that they both exhibited low IC_50_ values. Albendazole also exhibited a low IC_50,_ however, it was much slower acting, showing an effect only after 72 hrs at the highest concentration. As expected, quinine had no effect and thus an IC_50_ value could not be calculated.

**Table 1:** **IC**_**50 **_**values of Newly excysted larvae (NEL) and adult *E. caproni *following treatment with praziquantel, tribendimidine, albendazole and quinine**

**Drug**	**NEL assay**		**Adult worm assay**	
	**IC**_**50 **_**(μg/ml)**	**R- value**	**IC**_**50 **_**(μg/ml)**	**R- value**
Praziquantel	0.19	0.94	0.06	0.77
Tribendimidine	0.35	0.93	2.93	0.91
Albendazole	2.03	0.99	26.71	0.82
Quinine	n.d.	n.d.	n.d.	n.d.

IC_50_ values were calculated 72 hrs post exposure. R-value represents the goodness of fit. N.d. means the data for that drug did not produce a curve from which an IC_50_ value could be derived.

**Figure 2:**
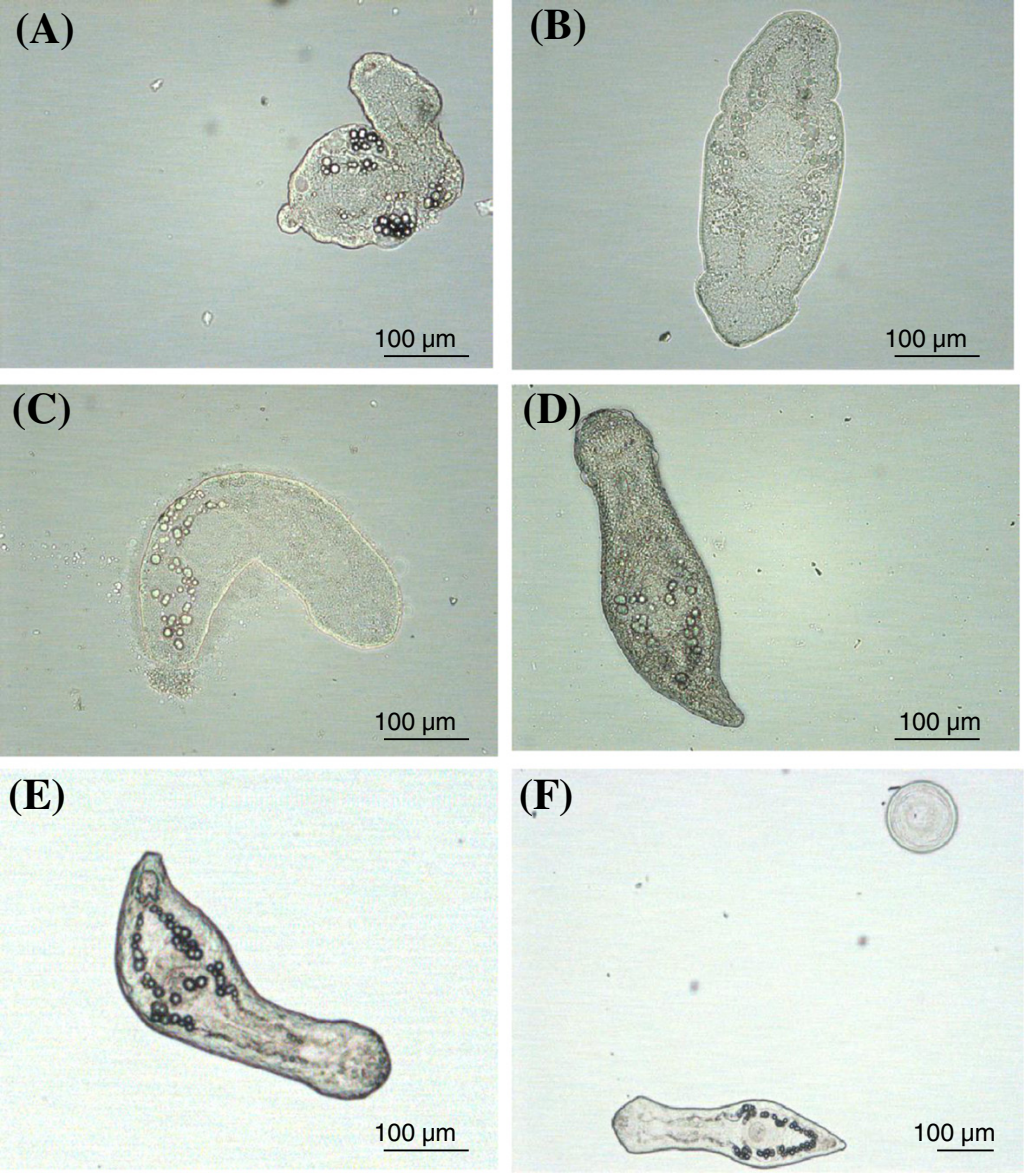
**Morphological effects of praziquantel, tribendimidine, albendazole and quinine on Newly excysted larvae (NEL).** Pictures taken after 72 hrs of exposure to **(A)** praziquantel **(B)** tribendimidine, **(C)** albendazole and **(D)** quinine, at a concentration of 30 μg/ml for tribendimidine, albendazole and quinine and 10 μg/ml for praziquantel. Image **(E)** shows a healthy NEL cultured in medium only. The bubbles surrounding the NEL in the albendazole image are an artifact due to the NEL being pressed by the glass slip and not due to drug effect. Figure **(F)** shows a NEL from the control well and an empty metacercarial shell.

The morphological effects of these compounds at the highest concentration (30 μg/ml) were visualized at 72 hrs post-exposure and are shown in Figure 2. Praziquantel caused severe tegumental damage and curling. Tribendimidine caused no visible tegumental damage, but rather a flattening of the larvae, perhaps due to paralysis. Albendazole caused slight distortions to the larval body, but its major impact was a reduction in motility. Quinine had no effect on the larval morphology or motility.

### Adult drug sensitivity assay

Table 1 shows the derived IC_50_ values from the adult worm assay at 72 hrs post-exposure. As in the NEL-based assay, praziquantel and tribendimidine presented low IC_50_ values, though the IC_50_ for tribendimidine was slightly higher for adult worms as compared to NEL. Both of these compounds were highly active at 30 μg/ml within 24 hrs of exposure.

**Figure 3:**
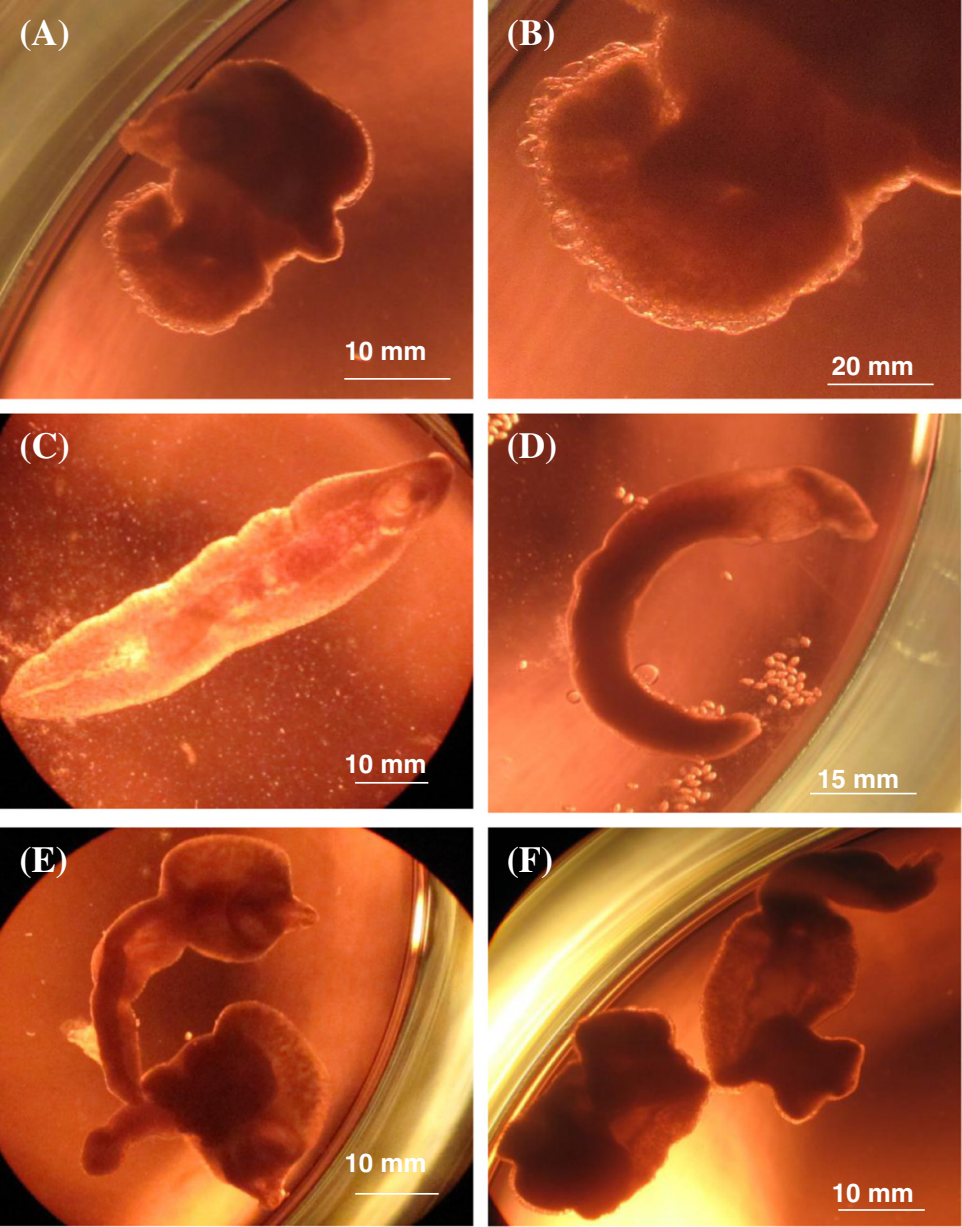
**Morphological effects of praziquantel, tribendimidine, albendazole and quinine on adult *E. caproni*.** Worms were incubated for 72 hrs with 30 μg/ml **(A)** praziquantel, **(C)** tribendimidine, **(D)** albendazole, **(E)** quinine or **(F)** medium only. The dots in **(D)** are eggs that were excreted by the adult worm. Figure **(B)** shows a portion of the adult worm from **(A),** zoomed in 2× to show the “blebbing” caused by praziquantel more visibly.

Similarly as carried out with the NEL, adult worms were imaged after 72 hrs of drug exposure at 30 μg/ml (Figure 3). The morphological effects of the drugs on adult worms were parallel to effects on NEL. Praziquantel caused severe tegument damage and curling. Tribendimidine had a paralyzing effect, where the worms were flattened but with minimal or no damage to the tegument. The main effect of albendazole was a decrease in the motility of the worm at 72 hrs, though in some worms, a small “blebbing” of the tegument could be observed. Quinine had no effect.

### Colorimetric markers

#### Resazurin

Fluorescence emissions (with background emission subtracted) of NEL incubated with resazurin reached a range of 516.7 (±183.0) to 737.3 (±156.9) at 10 min but failed to show a linear correlation to NEL number. After 20 min incubation time the signal dropped to below 0 and only increased marginally for all measurements up to 6 hrs of incubation. Furthermore, the signal did not correlate with the number of viable NEL per well and the signal from dead NEL was comparable. At 24 hrs incubation time, the signal levels were higher (range: 467.5 (± 88.3) at 20 NEL/well to 1720.0 ± 165.4 at 80 NEL/well) and reached a plateau at 60 NEL/well. Moreover, the signal from dead NEL was comparatively lower that that of live NEL (374.8 ± 195.2). However, at 24 hrs incubation time, the signal from the cyst only wells was high (1250 ± 18.3).

#### CellTiter-Glo® viability assay

Despite initial promising results, incubation of live NEL with CellTiter-Glo® failed to consistently produce a strong signal to NEL number correlation. The signal curves fluctuated wildly between measurements, though increasing the sensitivity of the reads (from1 read/well to 20 reads/well) on the SoftMax Pro® greatly diminished the between-read variation. Additionally, the signals generated from 100 dead NEL and 100 cysts were highly variable between measurements, sometimes producing a negligible signal and sometimes producing signals comparable to 20–40 live NEL.

#### Vybrant ® cytotoxicity assay

The fluorescence signals (background subtracted) of Vybrant® positively correlated with the number of dead NEL (R^2^ = 0.98) and reached a signal peak at 4 hrs, after which the signal levels continued to increase but the linearity of the curve started to decline. The signal plateaued at 80 NEL/well (6441.3 ± 0.5). However, the signal generated from 100 live NEL (1808.1 ± 951.8) was comparable to that of 40 dead NEL (2374.0 ± 615.5) and the signal generated from 100 cysts (4675.7 ± 312.4) was close to that of 60 dead NEL (4305.7 ± 1587.5).

## Discussion

Echinostomiasis may not always exhibit widespread national prevalence in endemic nations, however, local prevalence can be high, up to 65% in some areas [19]. Additionally, it is a stubbornly persistent disease due to continued consumption practices of uncooked freshwater fish, crustaceans or amphibians, socio-economic factors and the parasite’s broad host specificity [4,19]. Thus chemotherapy is essential to reducing infections, yet the reliance on only the broad-spectrum praziquantel as the sole treatment for echinostomiasis is ill-advised and the discovery of new active compounds is imperative [5,19]. Due to its convenient life-cycle maintenance and similarity to other intestinal food borne trematodes, *E. caproni* is a useful model for studies on intestinal food-borne trematodiasis [6]. *In vitro* drug sensitivity assays using adult *E. caproni* are effective, but are low-throughput, time-consuming and the required use of mice is expensive and ethically questionable. The aim of this study was to develop an effective *in vitro* drug sensitivity assay using NEL of *E. caproni.*

The first step was to test key parameters of the TBC excystation method. Optimal conditions for good excystation yields are summarized in Table 2, as determined by experiments in this study.

**Table 2:** Key factors for good (20–60%) excystation yields

**Excystation factor**	**Optimal values**
Cyst storage time	0–4 weeks
TBC medium:	
Trypsin concentration	1–5 mg/ml
Bile salts concentration	3–7 mg/ml
L-cysteine concentration	2–8 mg/ml
Amount of TBC medium	≥ 3 ml
Amount of cysts exposed to medium	No optimum
pH of TBC medium	7.8–8.2
Incubation temperature	39–41°C
Incubation time	1–2.5 hrs

Excystation rates were not affected in a dose-dependent manner by the concentrations of trypsin, bile salts or L-cystein, nor by the amount of TBC added or the number of cysts present in the TBC medium. Previous morphological studies show that the TBC medium marginally softens the shell of the metacercariae, but it is more so that the trypsin and bile salts, as well as the alkaline pH and warm temperature, are a trigger for the metacercariae to release enzymes that digest the shell from the inside [20]. Perhaps due to this latter point, we observe that the excystation rates are not exactly dose-dependent, but rather a threshold presence of trypsin and bile salt (as well as L-cysteine) is important.

In this study, it was shown that cysts stored in Locke’s 1:1 solution at 4°C should be kept no longer than 4 weeks, after which their viability significantly declines. This is in contrast to previous publications which allude that cysts can be stored in Locke’s 1:1 at 4°C for as long as 24 weeks post extraction [13]. Long storage times also make the cyst suspension more prone to fungal contaminations. The addition of 2% Amphotericin B to the medium did not resolve the matter because of its negative effect on metacercarial viability. However, unpublished observations indicate that filtering the Locke’s solution prior to use and changing the medium once a week almost eliminates the risk of contamination.

Other attempts to increase excystation rates failed. An acid-pepsin pre-treatment did not improve the excystation rate, but did not damage it either. Acid-pepsin accelerates excystation in the trematode *Clonorchis sinensis* as well as in other helminths [15,21]. However, this was not the case for *E. caproni*, suggesting that perhaps *in vivo* it is not a trigger either.

Throughout this study, excystation rates did not exceed an average of 60% (save for occasional outliers). This is in contrast to published rates of 50-100% [9,20,22]. There are several possibilities for this discrepancy. The first is that the composition of trypsin or the bile salts could play a role. Saxton *et al*. (2008), revealed that having to switch suppliers for trypsin and bile salts resulted in a significant drop in excystation rates, though this was remedied by the addition of L-cystein to their protocol [9,22]. Bile is not a homogenous and standardized mixture, containing a variety of bile acids in varying compositions. Moreover, some excystation publications for other helminths revealed that the bile salt source did in fact affect their excystation rates: for example, the use of cattle-derived bile salts versus the use of sheep-derived bile salts [22,23]. Other variations may entail the use of different reductants such as 0.02 M sodium dithionite [24] or simply a pre-treatment with only the reductant [25]. Applying a reductant has also been carried out in between an acid-pepsin pre-treatment and an alkaline trypsin-bile salt pre-treatment for other helminths [26].

The ability of NEL to survive for 5 days in 200 μl culture medium in a 96-well plate and the simplicity of their viability assessment facilitate their use in drug sensitivity assays. NEL can be incubated at a temperature of 37°C, 5% CO_2,_ in supplemented RPMI, Basch medium or Med 199.

Drug sensitivity assays using NEL incubated with praziquantel, tribendimidine, albendazole and quinine showed parallel results to adult worm assays. That is, praziquantel and tribendimidine were highly effective drugs against both stages, whereas albendazole was less effective and quinine was not effective at all. NEL are slightly more sensitive to the compounds, as indicated in Table 1. First, praziquantel and tribendimidine were highly effective against both stages of the worm, however, whereas they were not completely lethal to adult worms at a concentration of 30 μg/ml, at the same concentration, they were almost immediately lethal to NEL. Furthermore, although praziquantel and tribendimidine had similar IC_50_ values in NEL assays as in adult worm assays, the IC_50_ for tribendimidine in adult worms was higher. NEL were also far more sensitive to albendazole after 72 hrs, as opposed to adult worms whose viability was only reduced by a half at this time-point. This trend, where the larval stage is slightly more sensitive to drug effects than the adult stage, has also been observed for other helminths: L1 larvae of *T. muris* are more sensitive than the adult worms [27] and NTS of *S. mansoni* are mostly more sensitive than the adult *S. mansoni*[12]. The increased sensitivity of NEL renders them a favorable compound pre-screening tool in drug screening cascades, as compounds screened first on NEL are unlikely to produce false negatives.

Due to the slow and sometimes subjective nature of the microscopic read-out, an automated method using colorimetric markers would be a positive step forward towards developing a high-throughput drug-screening assay. However, none of the markers tested were adequate for use in an NEL drug sensitivity assay. CellTiter-Glo® failed to consistently produce a strong signal to NEL number curve. Resazurin produced a good signal to NEL number curve only after a long incubation time of 24 hrs but cyst-only control wells also produced high signals. Vybrant® did produce a good curve with a short incubation time of 4 hrs and a plateau was achieved at 80 NEL per well. However, here too, wells containing cysts only also produced a high signal. This is problematic as the excystation procedure does not trigger 100% of the metacercariae to excyst and currently, there is no practical way to separate cysts from NEL. Until we can do that, markers producing signals from cysts are not a consideration for use in drug sensitivity tests with NEL. Nonetheless, the observation that incubation with both resazurin and Vybrant® yielded high fluorescence signals from cysts is intriguing, suggesting some sort of metabolic or at least reducing activity in the cysts. It is worth noting that in order to place exact numbers of NEL for these assays, the larvae have to be manually counted in, as currently there is no short-hand method of determining their concentration to an exactitude that marker assays require. This is not only time consuming but it also makes for a non-sterile set-up, which can interfere with marker signals. Thus, it is likely that this issue would have to be resolved before further testing with markers can ensue.

## Conclusion

Here we have presented an alternative to the adult *E. caproni* drug sensitivity assay. The use of NEL instead of adult worms is faster, cheaper, higher throughput and more ethical. The results from the NEL and adult *in vitro* assays are highly comparable. NEL are even slightly more sensitive to the test compounds. This latter point makes an NEL-based assay an attractive pre-screening tool, as it would be unlikely to yield false negatives and therefore unlikely to reject active compounds in the screening cascade.

Nonetheless, improvements to the assay should be investigated. The excystation rates of the *E. caproni* metacercariae could be ameliorated. Furthermore, practicalities in assay set up, especially in separating cysts from NEL and accurately determining NEL concentration in a solution should be improved. Finally, in order to increase its potential as a high-throughput assay, further automated read-out methods should be researched.

## Competing interests

The authors declare that they have no competing interests.

## Authors’ contributions

GP, KI and JK designed the studies. GP carried out the experiments and wrote the first draft of the manuscript. KI and JK revised the manuscript. All authors read and approved the final version of the manuscript.
